# Supplementary material for: MetaQTL: a package of new computational methods for the meta-analysis of QTL mapping experiments
Source: BMC Bioinformatics. 2007 Feb 8;8:49. doi: 10.1186/1471-2105-8-49 (PMC1808479; doi:10.1186/1471-2105-8-49)
Supplement: Additional File 7 — MetaQTL Package : jar file and tutorial. This Zip archive contains both the MetaQTL JAR file and the files of the tutorial. [file 1471-2105-8-49-S7.zip › org.inra.metaqtl/doc/index-files/index-19.html]

T-Index


|  |  |  |
| --- | --- | --- |
| **Overview** \| Package \| Class \| Use \| **Tree** \| **Deprecated** \| **Index** \| **Help** | |  |
| **PREV LETTER**   **NEXT LETTER** | **FRAMES**    **NO FRAMES**     **All Classes** |  |


A B C D E F G H I K L M N O P Q R S T U V W X Y Z 

---


## **T**

**TEXT\_WIDTH** - Static variable in class org.metaqtl.graph.QtlLayer: The default width of the Text attribute of the Qtl **theta** - Variable in class org.metaqtl.MultiFitResult: The parameter estimates. **TICK\_CEX\_HEIGHT** - Static variable in class org.metaqtl.graph.QtlLayer: The default cex parameter for the tick height of the Qtl **TICK\_CEX\_WIDTH** - Static variable in class org.metaqtl.graph.QtlLayer: The default cex parameter for the tick width of the Qtl **TINY\_MAPPING\_DISTANCE** - Static variable in interface org.metaqtl.IMetaQtlConstants: The minimal distance in morgan **TINY\_MAPPING\_VARIANCE** - Static variable in interface org.metaqtl.IMetaQtlConstants: the minimal variance in morgan **TINY\_Z\_PROBA** - Static variable in class org.metaqtl.algo.EMAlgorithm: The minimal value for Z proba in EM-clustering **to** - Variable in class org.metaqtl.Qtl.QTLSupport: The position from the first marker to the right border of the IC. **toChromosome(IBioLGroup, IBioCross, int, int)** - Static method in class org.metaqtl.adapter.ChromosomeAdapter: This method converts a linkage group into a chromosome of type `Chromosome` with the given cross design and the given mapping context. **toChromosomes(IBioGenome)** - Static method in class org.metaqtl.adapter.ChromosomeAdapter: This methods converts the given map into an array of `Chromosome` which represents the chromosomes of the input mpe. **toEntity(BioEntityBean, IBioEntity)** - Static method in class org.thalia.bio.entity.adapter.BioEntityAdapter: **toEntity(Object)** - Method in class org.thalia.bio.entity.adapter.BioEntityCollectionAdapter: **toEntity(Object)** - Method in class org.thalia.bio.entity.adapter.GeneticMapBeanAdapter: **toEntity(Object)** - Method in class org.thalia.bio.entity.adapter.LGroupBeanAdapter: **toEntity(Object)** - Method in class org.thalia.bio.entity.adapter.LocusBeanAdapter: **toEntity(Object)** - Method in class org.thalia.bio.entity.adapter.OntologyBeanAdapter: **toEntity(Object)** - Method in class org.thalia.bio.entity.adapter.OntologyTermBeanAdapter: **toEntity(Object)** - Method in interface org.thalia.bio.IBioAdapter: Convert the given bio entity object into another compliant object. **toIBioGenome(Chromosome[], int)** - Static method in class org.metaqtl.adapter.ChromosomeAdapter: This routine converts an array of chromosomes into a genetic map with as many chromosomes as the length of the array. **toIBioGenome(MetaMap)** - Static method in class org.metaqtl.adapter.MetaMapAdapter: This methos convert a `MetaMap` object into an object which is consistent with the `IBioGenome` interface. **toQTL(IBioLocus)** - Static method in class org.metaqtl.adapter.QtlAdapter: This method converts an object which class implements the `IBioLocus interface into a Qtl. toString() - Method in class org.metaqtl.Chromosome: toString() - Method in class org.metaqtl.MultiFitResult: toString() - Method in class org.thalia.bio.entity.LGroup: totd - Variable in class org.metaqtl.Chromosome: The total distance of the chromosome. trait - Variable in class org.metaqtl.MetaQtlResult: The name of the trait. trait - Variable in class org.metaqtl.Qtl: The QTL trait data. traitNames - Variable in class org.metaqtl.MetaQtlModel: The trait names per chromosome. transformY(double) - Method in class org.metaqtl.graph.ChromAxe: Do a affine transformation of the given position d which is assumed to be in [0,dmax] in order to have its representation in the chromosome axe. tree - Variable in class org.metaqtl.MetaQtlResult: The qtl tree tree - Variable in class org.metaqtl.QtlPartition: The Qtl tree. Tree - Class in org.metaqtl: This class defines a tree. Tree() - Constructor for class org.metaqtl.Tree: Tree(TreeNode) - Constructor for class org.metaqtl.Tree: tree_method - Variable in class org.metaqtl.algo.QtlTreeAlgorithm: This global variable defines the method used by the aglomerative hierarchical clustering. TREE_NODE_WIDTH - Static variable in class org.metaqtl.graph.MetaGraphPar: TreeFactory - Class in org.metaqtl.factory: A factory to deal with distance trees. TreeFactory() - Constructor for class org.metaqtl.factory.TreeFactory: TreeNode - Class in org.metaqtl: This class represents a node in a binary Tree obtained when applying usual hirarchical clustering methods. TreeNode(int) - Constructor for class org.metaqtl.TreeNode: Creates a new instance of TreeNode with the given indice idx. TreeNode(int, TreeNode[]) - Constructor for class org.metaqtl.TreeNode: Creates a new instance of TreeNode with the given indice idx and the children of the node. TreeNode() - Constructor for class org.metaqtl.TreeNode: Creates a new instance of TreeNode with default attribute values. type - Variable in class org.metaqtl.Qtl.QTLCross: The type of the cross. type - Variable in class org.thalia.bio.entity.bean.LocusBean: type - Static variable in class org.thalia.bio.util.BioCrossTypes: TypesString - Static variable in interface org.thalia.bio.IBioLocus: typeToString(int) - Static method in class org.thalia.bio.util.BioLocusTypes`

---


|  |  |  |
| --- | --- | --- |
| **Overview** \| Package \| Class \| Use \| **Tree** \| **Deprecated** \| **Index** \| **Help** | |  |
| **PREV LETTER**   **NEXT LETTER** | **FRAMES**    **NO FRAMES**     **All Classes** |  |


A B C D E F G H I K L M N O P Q R S T U V W X Y Z 

---
